# Association of gain-of-function *EPHX2* polymorphism Lys55Arg with acute kidney injury following cardiac surgery

**DOI:** 10.1371/journal.pone.0175292

**Published:** 2017-05-26

**Authors:** Megan M. Shuey, Frederic T. Billings, Shouzou Wei, Ginger L. Milne, Hui Nian, Chang Yu, Nancy J. Brown

**Affiliations:** 1 Department of Pharmacology, Vanderbilt University School of Medicine, Nashville, Tennessee, United States of America; 2 Department of Anesthesiology, Vanderbilt University School of Medicine and Vanderbilt University Medical Center, Nashville, Tennessee, United States of America; 3 Department of Medicine, Vanderbilt University School of Medicine and Vanderbilt University Medical Center, Nashville, Tennessee, United States of America; 4 Department of Biostatistics, Vanderbilt University School of Medicine and Vanderbilt University Medical Center, Nashville, Tennessee, United States of America; University of Sao Paulo Medical School, BRAZIL

## Abstract

Twenty to thirty percent of patients undergoing cardiac surgery develop acute kidney injury (AKI). In mice, inhibition of soluble epoxide hydrolase (sEH) attenuates renal injury following ischemia-reperfusion. We tested the hypothesis that functional variants of *EPHX2*, encoding sEH, are associated with AKI after cardiac surgery. We genotyped patients in two independent cardiac surgery cohorts for functional *EPHX2* polymorphisms, Lys55Arg and Arg287Gln, and determined AKI using Acute Kidney Injury Network criteria. The 287Gln variant was not associated with AKI. In the discovery cohort, the gain-of-function 55Arg variant was associated with an increased incidence of AKI in univariate (p = 0.03) and multivariable (p = 0.04) analyses. In white patients without chronic kidney disease (CKD), the 55Arg variant was independently associated with AKI with an OR of 2.04 (95% CI 0.95–4.42) for 55Arg heterozygotes and 31.53 (1.57–633.19) for homozygotes (p = 0.02), after controlling for age, sex, body mass index, baseline estimated glomerular filtration rate, and use of cardiopulmonary bypass. These findings were replicated in the second cardiac surgery cohort. 12,13- and total- dihydroxyoctadecanoic acids (DiHOME): epoxyoctadecanoic acids (EpOME) ratios were increased in *EPHX2* 55Arg variant carriers, consistent with increased hydrolase activity. The *EPHX2* Lys55Arg polymorphism is associated with AKI following cardiac surgery in patients without preexisting CKD. Pharmacological strategies to decrease sEH activity might decrease postoperative AKI.

## Introduction

Twenty to thirty percent of patients undergoing cardiac surgery develop acute kidney injury (AKI) [1]. AKI following cardiac surgery is associated with a five-fold increase in postoperative mortality independent of other risk factors for early death [2–5] and leads to increased treatment costs and length of stay [6]. The development of AKI is influenced by both environmental factors [7] and genetic factors [8].

Renal ischemia-reperfusion (IR) injury, oxidative stress, inflammation, emboli, and neurohormonal activation contribute to the pathophysiology of AKI following cardiac surgery [9]. Epoxyeicosatrienoic acids (EETs), cytochrome P450 metabolites of arachidonic acid, oppose many of these processes. For example, EETs regulate renal blood flow by dilating afferent arterioles [10] and exert cellular and vascular anti-inflammatory [11], proliferative [12, 13], antithrombotic [14], and fibrinolytic [15] effects. Factors that influence EET concentrations or action may therefore affect the risk of AKI following cardiac surgery and may represent modifiable targets for the development of novel therapeutics. The enzyme soluble epoxide hydrolase (sEH) hydrolyzes bioactive EETs to less active dihydroxyeicosatrienoic acids (DHETs) [14]. Therefore, inhibiting sEH has the potential to increase EET levels and may affect an individual’s risk of AKI.

The potential for targeting sEH to affect AKI risk was demonstrated in a murine model of IR injury, in which administration of a sEH inhibitor prior to IR attenuated renal functional decline, tubular necrosis, and renal inflammation compared to vehicle, and the severity of IR-induced renal damage correlated inversely with endogenous EET levels [16]. To study whether sEH activity is associated with AKI in humans, we used a candidate gene approach focused on two known functional polymorphisms of *EPHX2*, the gene encoding sEH: a gain-of-function polymorphism (rs41507953 or Lys55Arg) and a loss-of-function polymorphism (rs751141 or Arg287Gln) [17]. We hypothesized that the gain-of-function polymorphism, rs41507953, would be associated with an increased incidence of AKI following cardiac surgery while the loss-of-function polymorphism, rs751141, would be associated with decreased incidence of AKI.

## Methods

### Study cohorts

Four hundred fifty-nine patients from the Statin AKI Cardiac Surgery RCT comprised the discovery cohort [18]. This trial included adult patients scheduled for elective cardiac surgery recruited from November 2009 to October 2014. Exclusion criteria included statin intolerance, acute coronary syndrome, liver dysfunction, and end stage renal disease. Subjects were randomized to perioperative atorvastatin or placebo to reduce the incidence of AKI (NCT00791648). Blood for DNA isolation was collected following induction of anesthesia but prior to surgery, and serum creatinine was measured prior to surgery and daily following surgery to calculate AKI. In 33 patients, blood was collected to measure EETs, DHETs, epoxyoctadecanoic acids (EpOMEs), and dihydroxyoctadecanoic acids (DiHOMEs) at three time points—prior to surgery, at the end of cardiopulmonary bypass or off-pump coronary artery bypass surgery, and at 9:00 on the morning of the first postoperative day.

Five hundred eighty-five patients from the Vanderbilt Cardiac Surgery Registry comprised the replication cohort [19]. The Registry was developed as a repository of clinical and laboratory data for use in outcome studies. Patients older than 18 years undergoing elective cardiac surgery at Vanderbilt University Medical Center were prospectively enrolled from November 1999 until November 2004 and provided blood for genomic DNA extraction. We included white patients in whom serum creatinine had been measured prior to surgery and on the second or third postoperative day following surgery. We excluded patients with end-stage renal disease on dialysis, patients who had a history of kidney transplantation, and with a baseline eGFR < 60 mL/min/m^2^ prior to surgery based on our findings in the discovery cohort (S1 Table).

Baseline glomerular filtration rate was estimated using the Chronic Kidney Disease Epidemiology Collaboration (CKD-EPI) equation with adjustment for gender and ethnicity [20]. AKI was defined using AKIN criteria [21], as an increase in serum creatinine >50% or by 0.3 mg/dL above baseline within 72 hours of surgery. Baseline creatinine was measured from plasma collected prior to surgery. Urine output criteria were not used to diagnose AKI following cardiac surgery due to the high prevalence of diuretic use in the early postoperative period and frequent perioperative intravascular hypovolemia, both of which confound the association between postoperative urine output and kidney injury [21].

Study protocols were approved by the Vanderbilt Institutional Review Board, and all participants provided written informed consent before enrollment.

### *EPHX2* genotyping

Genomic DNA was extracted from whole blood using the AutoPure LS extraction system (Qiagen, Valencia, CA, USA) following the manufacturer’s guidelines. *EPHX2* SNPs rs751141 (Arg287Gln) and rs41507953 (Lys55Arg) were genotyped using Applied Biosystems (Applied Biosystems, Foster City, CA, USA) TaqMan assays, and the assay ids are C___2045398_20 and C__32297897_10, respectively. All reactions were carried out at a volume of 5 μls in 384-well plates containing 0.125 μl 20x TaqMan assay, 5ng of genomic DNA and TaqMan Universal PCR Mastermix following the manufacturer’s guidelines. Products were scanned on the ABI 7900HT (Applied Biosystems) instrument equipped with SDS v2.4 (Applied Biosystems) for the creation of cluster plots and the identification of sample-associated fluorescent markers for determination of genotype call [22]. All genotyping was completed without knowledge of subject’s AKI status. The discovery cohort was genotyped for both the rs41507953 and rs751141 variants. The replication cohort was genotyped for the rs41507953 variant.

### Laboratory analyses

Creatinine was measured in plasma collected preoperatively and daily until discharge in the Clinical Laboratory Improvement Amendments (CLIA)-certified Vanderbilt Clinical Laboratory using an automated Jaffe reaction.

Blood was collected from subjects into citrate-containing phlebotomy tubes that also contained 2–3 mg triphenylphosphine to inhibit oxidation, immediately placed on ice, and then centrifuged at 1000 g at 0°C for 20 minutes to separate plasma. Following plasma separation, an additional 2 to 3 mg of triphenylphosphine were added to all plasma samples prior to storage at -80°C until analyte quantification.

Plasma EETs, DHETs, EpOMEs, and DiHOMEs were quantified by ultra-performance liquid chromatography/tandem mass spectrometry (UPLC/MS/MS), as follows. Deuterated internal standards (Cayman Chemical, Ann Arbor, MI, USA) were added to each sample. For lipids extraction, 3 ml 0.15M KCL was added to 1 ml plasma and extracted with acidified 8ml chloroform/methanol (CHCL3/CH3OH) mixture at a 2:1 ratio [23]. The organic phase was collected and saponified (0.4M KOH in 80% CH3OH). The samples’ EETs, EpOMEs, DHETs, and DiHOMEs were separated by SiO2 chromatography. SiO2-purified EETs and EpOMEs were hydrated to DHETs and DiHOMEs, respectively, in 9.5 M acetic acid (HOAc). EETs, EpOMEs, DHETs, and DiHOMEs were analyzed by UPLC/MS/MS. The UPLC was equipped with an Acquity BEH C18 column 1.0 x 100 mm, 1.7 μm, (Waters). To separate analytes, we performed a gradient elution starting with a 70% mobile phase A (15 mM ammonium acetate, pH 8.5) proceeding to a 60% mobile phase B (acetonitrile) over a period of five minutes. Following gradient elution, columns were washed for one minute with mobile phase B. The UPLC effluent was then subjected to negative ion electrospray ionization using a triple quadropule mass spectrometer, TSQ Quantum Vantage (Thermo Scientific, Hudson, NH, USA), equipped with an electrospray source operating in negative ion-mode. Analytes and internal standards were identified and quantified using selected reaction monitoring [24].

### Statistical analysis

There was no *a priori* statistical power calculation performed for determination of cohort size. Data are presented as frequencies for categorical variables and median with inner quartile range for continuous variables. Between-group comparisons were made using a chi-square test for categorical variables and Wilcoxon rank-sum test for continuous variables. We fit a logistic regression model for AKI on *EPHX2* genotype, as a two-level or three-level categorical variable, as well as baseline eGFR, age, sex, race, history of diabetes, BMI, and use of cardiopulmonary bypass. The variables used in the logistic regression model for AKI on *EPHX2* genotype were defined *a priori* based on literature [4, 25]. Results are reported as odds ratios and 95% confidence intervals. Mixed-effects models were fit for DiHOME:EpOME and EETs measured prior to surgery, at the end of cardiopulmonary bypass or off-pump coronary artery bypass surgery, and at 9:00 am on the morning of the first postoperative day with fixed effects of genotype, time, as well as interaction between genotype and time, with a random subject effect. The interaction term, when insignificant, was then removed from the model. Nominal two-sided p-values are reported and p-values ≤ 0.05 were considered statistically significant. We conducted 5 comparisons in the discovery cohort each at a 0.05 level providing a type-I error rate of 0.226. We have the same type-I error rate in our validation cohort. We only report significant results in the discovery cohort that are also significant in the validation cohort at a 0.05 level. Therefore, based on study design the overall type-I error rate is equivalent to 0.051 the square of the type-I error rates.

## Results

### Discovery cohort characteristics

The discovery cohort included 459 of the 615 patients enrolled in the Statin AKI Cardiac Surgery RCT who consented to provide DNA [18]. We did not collect DNA in 156 of the 615 patients in order to concentrate investigator efforts on recruitment, treatment adherence, and outcomes assessments. These 156 patients were similar to the 459 patients from whom we collected DNA (S2 Table). One hundred twelve patients (24.4%) in the discovery cohort developed AKI. Ninety-two of these 112 patients (82.1%) developed stage one AKI, nine patients (8.0%) stage two AKI, and eleven patients (9.8%) developed stage three AKI. Fifty-three of the 112 AKI patients (47.3%) had non-resolving AKI defined as a serum creatinine concentration still rising 72 hours after surgery. Seven patients (1.5%) required postoperative dialysis and five patients (1.1%) died following surgery. AKI patients were older, had higher baseline body mass index (BMI), had a lower baseline estimated glomerular filtration rate (eGFR), and were more likely to be diabetic than non-AKI patients (Table 1). Perioperative statin treatment did not reduce AKI [18].

**Table 1:** Characteristics of the discovery cohort according to acute kidney injury (AKI) development.

Characteristic	AKI (n = 112)	No AKI (n = 347)	p-value
Age, years	70.0 (63.0–77.0)	65.0 (56.0–74.0)	0.01
Female, n (%)	28 (25.0%)	116 (33.4%)	0.10
Black race, n (%)	6 (5.4%)	15 (4.3%)	0.65
BMI, kg/m^2^	29.2 (25.8–33.0)	27.3 (24.3–31.0)	<0.001
eGFR, mL/min/1.73m^2^	56.9 (42.3–78.3)	75.5 (59.7–89.2)	<0.001
Diabetes, n (%)	48 (42.9%)	101 (29.1%)	0.01
Congestive heart failure, n (%)	61 (54.5%)	123 (35.4%)	<0.001
Atrial fibrillation, n (%)	33 (29.5%)	80 (23.1%)	0.21
Systolic blood pressure, mmHg	131.0 (118.0–142.5)	127.0 (115.0–140.8)	0.79
Procedure characteristics			
CABG, n (%)	64 (57.1%)	158 (45.5%)	0.04
Valve surgery, n (%)	73 (65.2%)	229 (66.0%)	0.91
CPB use, n (%)	85 (75.9%)	245 (70.6%)	0.28
CPB duration, min	166.0 (109.5–199.5)	133.5 (102.3–173.7)	0.27
Cross clamp use, n (%)	65 (58.0%)	154 (44.4%)	0.03
Cross clamp duration, min	118.0 (76.0–150.0)	92 (68.0–125.0)	0.04
Genotype			
Lys55Arg, additive			0.03
Lys/Lys, n (%)	81 (72.3%)	288 (83.0%)	
Lys/Arg, n (%)	28 (25.0%)	56 (16.1%)	
Arg/Arg, n (%)	3 (2.7%)	3 (0.9%)	
Lys55Arg, dominant			0.01
Lys/Lys, n (%)	81 (72.3%)	288 (83.0%)	
Lys/Arg, Arg/Arg, n (%)	31 (27.7%)	59 (17.0%)	
Arg287Gln, additive			0.41
Arg/Arg, n (%)	95 (77.9%)	283 (82.0%)	
Arg/Gln, n (%)	17 (15.1%)	57 (16.5%)	
Gln/Gln, n (%)	0 (0.0%)	5 (1.5%)	
Arg287Gln, dominant			0.57
Arg/Arg, n (%)	95 (77.9%)	283 (82%)	
Arg/Gln, Gln/Gln, n (%)	17 (15.1%)	62 (18%)	

Data are presented as median (interquartile range) unless otherwise indicated.

Abbreviations: AKI, acute kidney injury; BMI, body mass index; eGFR, estimated glomerular filtration rate; CABG, coronary artery bypass grafting; CPB, cardio-pulmonary bypass; min, minutes

### *EPHX2* genotype and incidence of AKI, discovery cohort

*EPHX2* Lys55Arg and Arg287Gln genotypes were in Hardy-Weinberg equilibrium. The distribution of Lys55Arg and Arg287Gln genotypes were Lys/Lys:Lys/Arg:Arg/Arg = 369:84:6 and Arg/Arg:Arg/Gln:Gln/Gln = 378:74:5, respectively. The minor allele frequencies were 10.7% and 9.2%, respectively, consistent with frequencies reported previously by the Exome Aggregation Consortium (ExAC) [26]. Further, the frequency of the *EPHX2* 55Arg allele was higher in black patients, 16.7%, compared to white patients, 10.2%, also consistent with frequencies reported in ExAC.

In a dominant genetic model of inheritance (Arg/Arg+Lys/Arg vs. Lys/Lys), the incidence of AKI was significantly increased in patients carrying the gain-of-function 55Arg variant of sEH, 34.4%, compared to 22% in non-carriers (p = 0.01, Table 1). Likewise, in an additive model of genetic inheritance (Lys/Lys vs. Lys/Arg and Lys/Lys vs. Arg/Arg), the incidence of AKI was significantly higher for patients with the gain-of-function 55Arg variant. Fifty percent of 55Arg homozygous patients and 33.3% of heterozygous carriers developed AKI, compared to 22% in noncarriers (p = 0.03, Table 1). There was no association, however, between the Arg287Gln genotype and incident AKI (p = 0.41).

In a multivariable logistic regression model for AKI incidence that included Lys55Arg genotype, gender, black race, BMI, history of diabetes, baseline eGFR, and use of cardiopulmonary bypass (CPB), Lys55Arg genotype, gender, BMI, and baseline eGFR were significant independent predictors of postoperative AKI (Table 2). The logistic regression model has a Nagelkerke R^2^ = 0.19 and a C-index = 0.73. The gain-of-function 55Arg variant was independently associated with a 79% increase in the odds of AKI (CI: 1.04–3.09; p = 0.04) compared to Lys/Lys variant.

**Table 2:** Multivariable logistic regression model for acute kidney injury (AKI) in the discovery cohort adjusted for age, sex, race, body mass index, baseline estimated glomerular filtration rate, history of diabetes, and cardiopulmonary bypass graft use.

Variable	Odds Ratio	95% CI		p-value
Lys55Arg (Lys/Arg+Arg/Arg:Lys/Lys)	1.79	1.04	3.09	0.04

### Analysis in whites with and without chronic kidney disease

Because the proportion of blacks undergoing cardiac surgery in the discovery cohort was small and because the frequency of the 55Arg allele is increased in blacks compared to whites, we repeated the analyses in white patients alone. Because preexisting CKD is associated with an increased risk of AKI [27, 28], we also stratified by the presence or absence of CKD at baseline. Table 3 provides baseline characteristics of the discovery cohort following stratification. Patients with preexisting CKD experienced higher rates of AKI, 58.4% compared to 17.1% in those without preexisting CKD.

**Table 3:** Baseline characteristics of whites from the discovery cohort stratified by perioperative baseline eGFR.

	**eGFR < 60 mL/min/1.73m**^**2**^			**eGFR ≥ 60 mL/min/1.73m**^**2**^		
Characteristic	AKI (n = 73)	No AKI (n = 42)	p-value	AKI (n = 54)	No AKI (n = 261)	p-value
Age, years	71.5 (64.0–77.0)	71.0 (63.0–77.0)	0.78	69.5 (62.2–78.0)	64.0 (56.0–73.0)	0.01
Female, n (5)	16 (30.8%)	35 (47.9%)	0.05	12 (22.2%)	75 (28.7%)	0.33
BMI, kg/m2	29.8 (25.6–33.2)	27.7 (25.2–31.7)	0.22	28.9 (26.2–33.1)	26.9 (24.0–30.9)	0.002
eGFR, mL/min/1.73m^2^	45.3 (35.3–50.5)	47.8 (41.8–53.0)	0.05	79.5 (73.1–87.9)	82.4 (71.5–94.0)	0.46
CHF, n (%)	37 (66.1%)	38 (50.7%)	0.11	24 (44.4%)	85 (32.6%)	0.12
Atrial fibrillation, n(5)	19 (33.9%)	22 (29.3%)	0.70	14 (25.9%)	58 (21.3%)	0.60
Diabetes, n (%)	31 (59.6%)	25 (34.2%)	0.01	14 (25.9%)	71 (27.2%)	0.85
Procedure characteristics						
CABG, n(%)	33 (58.7%)	28 (37.3%)	0.84	31 (55.4%)	130 (47.8%)	0.31
Valve surgery, n (%)	42 (75.0%)	58 (77.3%)	0.84	31 (55.4%)	171 (62.9%)	0.30
CPB, n (%)	45 (86.5%)	59 (80.8%)	0.40	37 (68.5%)	180 (69.0%)	0.95
CPB duration, min	162 (111.0–193.0)	130 (109.5–163.8)	0.76	171.5 (107.8–254.3)	134.5 (101.3–177.0)	0.01
Cross clamp, n(%)	33 (45.21%)	35 (67.3%)	0.23	32 (59.3%)	119 (45.6%)	0.02
Cross clamp duration, min	118 (71.0–141.0)	93 (71.0–117.0)	0.18	120.5 (81.5–158.0)	89 (68.0–133.0)	0.07
Genotype						
Lys55Arg, additive			0.32			0.02
Lys/Lys, n (%)	39 (75.0%)	58 (79.0%)		39 (72.0%)	219 (84.0%)	
Lys/Arg, n (%)	13 (25.0%)	13 (18.0%)		13 (24%)	41 (16.0%)	
Arg/Arg, n (%)	0 (0.0%)	2 (3.0%)		2 (4.0%)	1 (0.0%)	
Lys55Arg, dominant			0.56			0.04
Lys/Lys, n (%)	39 (75.0%)	58 (79.0%)		39 (72.0%)	219 (84.0%)	
Lys/Arg,Arg/Arg, n (%)	13 (25.0%)	15 (21.0%)		15 (28.0%)	42 (16.0%)	
	**eGFR < 60 mL/min/1.73m**^**2**^			**eGFR ≥ 60 mL/min/1.73m**^**2**^		
Characteristic	AKI (n = 73)	No AKI (n = 42)	p-value	AKI (n = 54)	No AKI (n = 261)	p-value
Age, years	71.5 (64.0–77.0)	71.0 (63.0–77.0)	0.78	69.5 (62.2–78.0)	64.0 (56.0–73.0)	0.01
Female, n (5)	16 (30.8%)	35 (47.9%)	0.05	12 (22.2%)	75 (28.7%)	0.33
BMI, kg/m2	29.8 (25.6–33.2)	27.7 (25.2–31.7)	0.22	28.9 (26.2–33.1)	26.9 (24.0–30.9)	0.002
eGFR, mL/min/1.73m^2^	45.3 (35.3–50.5)	47.8 (41.8–53.0)	0.05	79.5 (73.1–87.9)	82.4 (71.5–94.0)	0.46
CHF, n (%)	37 (66.1%)	38 (50.7%)	0.11	24 (44.4%)	85 (32.6%)	0.12
Atrial fibrillation, n(5)	19 (33.9%)	22 (29.3%)	0.70	14 (25.9%)	58 (21.3%)	0.60
Diabetes, n (%)	31 (59.6%)	25 (34.2%)	0.01	14 (25.9%)	71 (27.2%)	0.85
Procedure characteristics						
CABG, n(%)	33 (58.7%)	28 (37.3%)	0.84	31 (55.4%)	130 (47.8%)	0.31
Valve surgery, n (%)	42 (75.0%)	58 (77.3%)	0.84	31 (55.4%)	171 (62.9%)	0.30
CPB, n (%)	45 (86.5%)	59 (80.8%)	0.40	37 (68.5%)	180 (69.0%)	0.95
CPB duration, min	162 (111.0–193.0)	130 (109.5–163.8)	0.76	171.5 (107.8–254.3)	134.5 (101.3–177.0)	0.01
Cross clamp, n(%)	33 (45.21%)	35 (67.3%)	0.23	32 (59.3%)	119 (45.6%)	0.02
Cross clamp duration, min	118 (71.0–141.0)	93 (71.0–117.0)	0.18	120.5 (81.5–158.0)	89 (68.0–133.0)	0.07
Genotype						
Lys55Arg, additive			0.32			0.02
Lys/Lys, n (%)	39 (75.0%)	58 (79.0%)		39 (72.0%)	219 (84.0%)	
Lys/Arg, n (%)	13 (25.0%)	13 (18.0%)		13 (24%)	41 (16.0%)	
Arg/Arg, n (%)	0 (0.0%)	2 (3.0%)		2 (4.0%)	1 (0.0%)	
Lys55Arg, dominant			0.56			0.04
Lys/Lys, n (%)	39 (75.0%)	58 (79.0%)		39 (72.0%)	219 (84.0%)	
Lys/Arg,Arg/Arg, n (%)	13 (25.0%)	15 (21.0%)		15 (28.0%)	42 (16.0%)	

Data are presented as median (interquartile range) unless otherwise indicated.

Abbreviations: AKI, acute kidney injury; BMI, body mass index; eGFR, estimated glomerular filtration rate; CHF, congestive heart failure; CABG, coronary artery bypass grafting; CPB, cardio-pulmonary bypass; min, minutes

In whites without preexisting CKD the incidence of AKI was significantly higher in carriers of the 55Arg allele compared to noncarriers under both dominant and additive genetic models, (p = 0.04 and p = 0.02, respectively) (Table 3). The *EPHX2* Arg55 genotype was not associated with AKI among whites with preexisting CKD in the discovery cohort. Interestingly BMI was also associated with AKI in patients without CKD but not those with CKD.

In a multivariable model of AKI incidence including Lys55Arg genotype, gender, BMI, history of diabetes, baseline eGFR, and use of CPB the Lys55Arg genotype independently predicted the development of postoperative AKI in whites without preexisting CKD (Table 4). The logistic regression model has a Nagelkerke R^2^ = 0.18 and a C-index = 0.74. The odds ratios for AKI in 55Arg heterozygotes and homozygotes were 2.04 (CI: 0.95–4.42) and 31.53 (CI: 1.57–633.19), respectively (p = 0.02 for overall genotype effect).

**Table 4:** Multivariable logistic regression model for acute kidney injury (AKI) in whites with baseline eGFR ≥ 60 mL/min/1.73m^2^ in the discovery cohort adjusted for age, sex, body mass index, baseline estimated glomerular filtration rate, history of diabetes, and cardiopulmonary bypass graft use.

Variable	Odds Ratio	95% CI		p-value
Lys55Arg (Lys/Lys)	1.00			0.02
(Lys/Arg)	2.04	0.95	4.42	
(Arg/Arg)	31.53	1.57	633.19	

### Replication cohort characteristics

The replication cohort consisted of 585 white patients without preexisting CKD from the Vanderbilt Cardiac Surgery Registry, a registry of patients undergoing coronary artery bypass graft with or without concurrent valve surgery from 1999–2004 who were consented for DNA collection and analysis [19]. The entire registry included 591 white patients without preexisting CKD, however, we were unable to determine genotype for six of these patients.

### *EPHX2* genotype and incidence of AKI, replication cohort

The Lys55Arg genotype exhibited Hardy-Weinberg equilibrium and the Lys55Arg genotype distribution was Lys/Lys:Lys/Arg:Arg/Arg = 475:106:4, with a minor allele frequency of 9.7%. Baseline patient characteristics in the replication cohort were similar to patient characteristics in the discovery cohort (Table 5). The incidence of AKI was 26%. Similar to the discovery cohort, individuals who developed AKI were older and heavier than those who did not develop AKI. In addition, there was a significant association between AKI and gender (p = 0.03) as well as history of diabetes (p = 0.02). Consistent with the discovery cohort, the incidence of AKI was significantly higher in carriers of the 55Arg allele compared to noncarriers under a dominant genetic model (p = 0.05) and under an additive (p = 0.03) (Table 5).

**Table 5:** Baseline characteristics of whites from the replication cohort with a baseline eGFR ≥ 60 mL/min/1.73m^2^.

Characteristic	AKI (n = 154)	No AKI (n = 431)	p-value
Age, years	58.1 (50.7–69.9)	58.0 (47.4–67.2)	0.04
Female, n (%)	37 (24.0%)	144 (33.4%)	0.03
BMI, kg/m^2^	28.3 (25.2–32.7)	27.8 (24.6–31.2)	0.04
eGFR, mL/min/1.73m^2^	83.6 (71.3–96.4)	86.6 (74.6–98.2)	0.14
Diabetes, n (%)	52 (33.8%)	103 (23.9%)	0.02
CHF, n (%)	45 (29.2%)	86 (20.0%)	0.06
Atrial Fibrillation, n (%)	26 (16.9%)	70 (16.2%)	0.73
Procedure characteristics			
CABG, n (%)	108 (70.1%)	283 (65.7%)	0.32
Valve surgery, n (%)	51 (33.1%)	150 (34.8%)	0.71
CPB, n (%)	149 (96.8%)	401 (93.0%)	0.10
CPB duration, min	124.5 (100.0–164.0)	115 (90.0–151.0)	0.24
Genotype			
Lys55Arg, additive			0.03
Lys/Lys, n (%)	117 (76.0%)	358 (83.0%)	
Lys/Arg, n (%)	34 (22.0%)	72 (17.0%)	
Arg/Arg, n (%)	3 (2.0%)	1 (0.0%)	
Lys55Arg, dominant			0.05
Lys/Lys, n (%)	117 (76.0%)	358 (83.0%)	
Lys/Arg, Arg/Arg, n (%)	37 (24.0%)	73 (17.0%)	

Data are presented as median (interquartile range) unless otherwise indicated.

Abbreviations: AKI, acute kidney injury; BMI, body mass index; eGFR, estimated glomerular filtration rate; CHF, congestive heart failure; CABG, coronary artery bypass grafting; CPB, cardio-pulmonary bypass; min, minutes

In a multivariable model of AKI incidence including Lys55Arg genotype, gender, BMI, history of diabetes, baseline eGFR, and use of CPB the Lys55Arg genotype independently predicted the development of postoperative AKI in whites without preexisting CKD in the replication cohort (Table 6). The logistic regression model has a Nagelkerke R^2^ = 0.09 and a C-index = 0.63. The odds ratios for AKI were 1.47 (CI: 0.90–2.37) for 55Arg heterozygotes and 11.28 (CI: 1.06–122.54) for 55Arg homozygotes (p = 0.05) for effect of genotype.

**Table 6:** Multivariable logistic regression model for acute kidney injury (AKI) in whites with baseline eGFR > 60 mL/min/1.73m^2^ in the replication cohort adjusted for age, sex, body mass index, baseline estimated glomerular filtration rate, history of diabetes, and cardiopulmonary bypass graft use.

Variable	Odds Ratio	95% CI		p-value
Lys55Arg (Lys/Lys)	1.00			0.05
(Lys/Arg)	1.47	0.90	2.37	
(Arg/Arg)	11.38	1.06	122.54	

### sEH activity and plasma EETs

**Fig 1:**
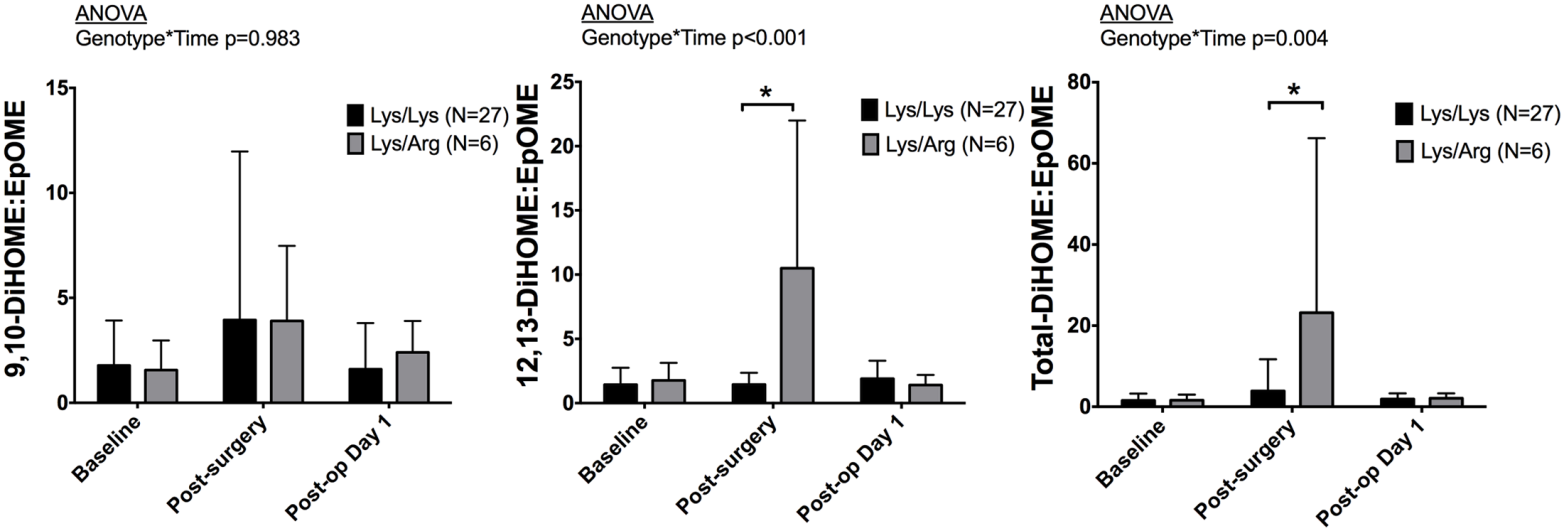
Dihydroxy-12Z-octadecenoic acids:epoxy-12Z-octadecenoic acids (DiHOME:EpOME) ratios in plasma from 33 patients collected before surgery, following surgery and on post-operative day one according to Lys55Arg genotype. By analysis of repeated measures, carriers of the 55Arg variant G allele had higher sEH activity over time than homozygous AA. *p<0.05 for post hoc comparison.

We collected blood from 33 consecutive patients in the discovery cohort to measure sEH activity and plasma concentrations of EETs prior to surgery, at the end of surgery, and on postoperative day 1. We calculated the molar ratio of DiHOME:EpOME in plasma to measure sEH activity, as these lipids are more stable and circulate at higher concentrations than EETs and DHETs [29, 30]. There was a significant genotype by time interaction for the 12,13- and total-DiHOME:EpOME ratios, p<0.001 and p = 0.004, respectively (Fig 1). 12,13- and total-DiHOME:EpOME ratios peaked at the end of surgery and returned to near baseline by postoperative day 1. In 55Arg carriers the median 12,13-DiHOME:EpOME ratio was 1.27 (interquartile range 1.19–1.39) at baseline, increased to 6.0 (5.8–9.9) at the end of surgery, and decreased to 1.25 (0.84–2.13) by postoperative day 1. A similar trend was noted in Lys/Lys patients: 0.98 (0.71–1.67) at baseline, 1.6 (1.1–2.1) at the end of surgery, and 1.26 (0.86–1.76) on postoperative day 1. The peak in the 12,13- and total-DiHOME:EpOME ratio was significantly higher in 55Arg carriers compared to noncarriers (p<0.001 and p<0.001, respectively, Fig 1).

Baseline total-, 12,13-, and 9,10- DiHOME:EpOME ratios were not significantly higher in the small number of patients in whom they were measured and who subsequently developed AKI (n = 6) versus those that did not develop AKI (n = 25) (S3 Table). Baseline plasma concentrations of 11,12-EETs were significantly lower in AKI versus non-AKI patients (p = 0.01, S3 Table).

## Discussion

We report for the first time that a gain-of-function variant of *EPHX2*, the gene encoding for sEH, is associated with AKI following cardiac surgery. In multivariable analyses in white patients without preexisting CKD, the development of AKI was associated with *EPHX2* Lys55Arg genotype in two separate cardiac surgery cohorts. In addition, we confirmed that sEH activity is increased in patients carrying the gain-of-function 55Arg allele *in vivo* in humans and found that baseline plasma levels of 11,12-EETs were significantly lower in patients who developed AKI.

Renal EETs have potent vasodilatory [10], proliferative [12, 13], anti-inflammatory [11], fibrinolytic [15], and antithrombotic [14] effects that may protect against AKI following IR injury, one of the mechanisms that leads to AKI after cardiac surgery. The beneficial effects of EETs are limited by their hydrolysis to the less active eicosanoids DHETs by the enzyme sEH [14]. The *EPHX2* polymorphism, Lys55Arg, was first identified in 2003 by Przybyla-Zawislak *et al* [17]. Using site-directed mutagenesis of human sEH expressed in whole insect cells, these scientists determined the 55Arg variant demonstrates increased hydrolase activity compared to the wild-type enzyme [17]. Subsequently, Srivastava *et al*. reported that the 55Arg variant also has decreased phosphatase activity and exhibits a significantly higher ratio of hydrolase to phosphatase activity [31].

Our findings in humans extend prior research in rodents suggesting that activation of sEH contributes to renal injury. In rodent models, sEH inhibition or genetic deletion of *EPHX2* attenuates renal damage due to hypertension [32, 33], salt-sensitive hypertension [34, 35], diabetes [36], and IR injury [16]. In humans, we have previously reported that the *EPHX2* 55Arg variant is associated with decreased endothelium-dependent vasodilation [37]. Decreased endothelial function reduces renal blood flow and leads to IR-induced AKI [38, 39]. Therefore, the gain-of-function *EPHX2* 55Arg variant may contribute to a reduction in endothelium-dependent renal vasodilation blood flow resulting in AKI following cardiac surgery.

The presence of CKD prior to surgery, however, may limit the protective effects of EETs because CKD is associated with endothelial dysfunction, even at the lowest levels of renal impairment [40, 41], and EETs are synthesized in the vascular endothelium [42]. Therefore CKD-associated endothelial dysfunction may decrease any protective effects of EETs, endothelial-derived hyperpolarizing factors [43, 44] that dilate renal vasculature. Diminished endothelial-dependent renal vasodilation in response to EETs due to CKD could account for the observation that Lys55Arg genotype was associated with AKI following cardiac surgery in patients with normal baseline renal function but not in those with CKD in both the discovery and replication cohorts.

Because preexisting CKD may mask the protective effects of EETs we conducted stratified analyses in patients with and without preexisting CKD. In previously published studies, the risk of AKI may be increased by as much as 10-fold in patients with CKD compared to in patients without CKD [27, 28, 45, 46]. In our discovery cohort we identified a similar increase in AKI risk in patients with preexisting CKD; 58.4% of patients with CKD developed AKI, compared to 17.1% without CKD. In addition, the reliability of AKIN criteria for the classification of AKI is decreased in patients with CKD [21, 47], further supporting conducting stratified analyses in patients with and without pre-existing CKD.

Because human sEH possesses both epoxide hydrolase and phosphatase activity [48, 49], we examined whether the *EPHX2* Lys55Arg polymorphism is associated with hydrolase activity *in vivo* in humans. We measured the ratio of plasma DiHOME:EpOME in 33 patients as a functional measure of sEH activity. Previous studies have supported the use of this ratio as a robust measure of sEH hydrolase activity *in vivo* because the linoleic acid metabolites, EpOME and DiHOME, are more stable and circulate at concentrations two orders of magnitude higher than that of their arachidonic acid counterparts, EETs and DHETEs [29, 30]. We found that sEH activity, quantified by either the 12,13-DiHOME:EpOME ratio or the total-DiHOME:EpOME ratio, was significantly increased in carriers of the 55Arg variant allele after surgery, confirming that *EPHX2* 55Arg is associated with an in increase in hydrolase activity in humans *in vivo*.

While sEH activity was higher in carriers of the 55Arg allele, plasma sEH activity increased during surgery regardless of Lys55Arg genotype. The mechanism by which sEH may be induced during surgery is not known. Previous work has shown plasma angiotensin II levels rise during CPB and remain elevated for up to four hours following surgery [50]. Angiotensin II is known to induce sEH expression [51], suggesting one potential mechanism for the observed increase in sEH activity.

The role of sEH phosphatase activity in renal injury is not well known. A recent study using an *EPHX2* murine knockout, demonstrated the loss of both epoxide hydrolase and phosphatase activity increases renal injury following IR [52]. This result contradicts the observation made in murine models of IR-induced renal injury treated with specific sEH inhibitors that target the catalytic pocket of sEH’s C domain responsible for sEH's epoxide hydrolase activity [53]. Modulation of the hydrolase activity of sEH is sufficient to affect renal injury after IR and future studies are needed to elucidate whether phosphatase activity plays a role, protective or other, in renal injury.

We did not find a significant association between the loss-of-function Arg287Gln genotype and the incidence of AKI or the activity of sEH. These results suggest that Arg287Gln is not associated with the development of AKI, but this could also reflect an inadequate sample size due to low frequency of the minor allele of this polymorphism. It is also possible that the effect of the Arg287Gln polymorphism depends on racial background. For example, we previously reported that the loss-of-function *EPHX2* 287Gln variant is associated with increased bradykinin-stimulated vasodilation in black Americans but not white Americans [37], and in the Coronary Artery Risk Development in Young Adults (CARDIA) study, the Arg287Gln polymorphism was associated with coronary artery calcification in blacks but not whites [54]. As noted earlier, the majority of patients undergoing cardiac surgery in both the discovery and the replication cohorts were white.

A limitation of this study is the use pre-determined sample sizes in both cohorts and a lack of a priori power calculations. Therefore, the study’s power is dependent on the allele frequencies and the strength of the association. Consequently as mentioned previously, potentially clinically meaningful associations may not meet the threshold for reporting. Nevertheless, this does not affect the validity of the detected association.

Further, the results of this study suggest that additional research to evaluate the potential clinical implications of sEH inhibitors to attenuate AKI warrants consideration. Inhibitors of sEH are under development and one inhibitor has been studied in phase II clinical trials for treatment of mild-to-moderate hypertension and impaired glucose tolerance [55–57]. The association between increased sEH activity and AKI identified in this study, coupled with a growing body of research supporting a protective effect of sEH inhibition in rodent models [32, 35, 36, 58, 59], suggests that pharmacologic inhibition of sEH should be tested in clinical trials to assess its effect on AKI in patients undergoing cardiac surgery.

## Supporting information

S1 Table Multivariable logistic regression model for acute kidney injury (AKI) in whites with baseline eGFR < 60 mL/min/1.73m^2^ in the discovery cohort. Abbreviations: BMI, body mass index; eGFR, estimated glomerular filtration rate; CPB, cardio-pulmonary bypass.
(PDF)

S2 Table Characteristics of the discovery cohort according to DNA availability. Data are presented as mean (95% confidence interval) unless otherwise indicated. Abbreviations: AKI, acute kidney injury; BMI, body mass index; eGFR, estimated glomerular filtration rate; CABG, coronary artery bypass grafting; CPB, cardio-pulmonary bypass; min, minutes.
(PDF)

S3 Table Baseline plasma DiHOME/EpOME ratio, a measure of soluble epoxide hydrolase activity, and epoxyeicosatrienoic acids (EETs) concentrations in patients who did or did not develop acute kidney injury (AKI). Data are presented as median (interquartile range) unless otherwise indicated.
(PDF)
