# Supplementary material for: Association of gain-of-function EPHX2 polymorphism Lys55Arg with acute kidney injury following cardiac surgery
Source: PLoS One. 2017 May 26;12(5):e0175292. doi: 10.1371/journal.pone.0175292 (PMC5446112; doi:10.1371/journal.pone.0175292)
Supplement: S1 Table — Abbreviations: BMI, body mass index; eGFR, estimated glomerular filtration rate; CPB, cardio-pulmonary bypass. (PDF) [file pone.0175292.s001.pdf]

S1 Table. Multivariable logistic regression model for acute kidney injury (AKI) in whites with baseline eGFR < 60 mL/min/1.73m^2 in the discovery cohort

Variable	Odds Ratio	95% CI		p-value
Lys55Arg (Lys/Lys)	1.00			0.79
(Lys/Arg)	1.35	<0.001	4.49	
(Arg/Arg)	<0.001	$2.46e^{-26}$	$1.89e^{19}$	
Age, years		0.74	1.11	0.34
Sex, female vs. male		0.14	0.79	0.01
BMI, kg/m^2		0.77	1.49	0.69
History of diabetes mellitus, yes vs. no		1.10	5.76	0.03
Baseline eGFR, mL/min/1.73 m^2		0.66	0.97	0.02
CPB use, yes vs. no		0.64	5.80	0.24

Abbreviations: BMI, body mass index; eGFR, estimated glomerular filtration rate; CPB, cardio-pulmonary bypass
