# Supplementary material for: Association of gain-of-function EPHX2 polymorphism Lys55Arg with acute kidney injury following cardiac surgery
Source: PLoS One. 2017 May 26;12(5):e0175292. doi: 10.1371/journal.pone.0175292 (PMC5446112; doi:10.1371/journal.pone.0175292)
Supplement: S2 Table — Data are presented as mean (95% confidence interval) unless otherwise indicated. Abbreviations: AKI, acute kidney injury; BMI, body mass index; eGFR, estimated glomerular filtration rate; CABG, coronary artery bypass grafting; CPB, cardio-pulmonary bypass; min, minutes. (PDF) [file pone.0175292.s002.pdf]

S2 Table. Characteristics of the discovery cohort according to DNA availability

Characteristic	DNA available (n=459)	DNA not available (n=156)	p-value
Acute kidney injury, n (%)	112 (24.4%)	31 (20.1%)	0.27
Age, years	65.6 (64.5 - 66.8)	65.2 (63.4 - 67.2)	0.77
Female, n (%)	144 (31.4%)	44 (28.2%)	0.48
Black race, n (%)	21 (4.6%)	5 (3.2%)	0.65
BMI, kg/m^2	56.8 (54.1 – 59.6)	56.8 (52.0 - 61.7)	1.00
eGFR, $mL/min/1.73m^2$	70.2 (68.1 - 72.2)	70.3 (66.6 - 73.9)	0.96
Diabetes, n (%)	150 (32.7%)	53 (34.0%)	0.77
Congestive heart failure, n (%)	185 (40.3%)	58 (37.2%)	0.51
Atrial fibrillation, n (%)	113 (24.6%)	28 (18.0%)	0.10
Systolic blood pressure, mmHg	130.2 (128.3 – 132.0)	130.0 (127.1 – 132.9)	0.93
Procedure characteristics			
CABG, n(%)	221 (48.1%)	79 (50.6%)	0.64
Valve surgery, n (%)	80 (17.4%)	24 (15.4%)	0.62
CPB use, n (%)	330 (71.9%)	105 (67.3%)	0.31
CPB duration, min	162.8 (149.1-176-6)	147.9 (130.6 – 165.2)	0.27
Cross clamp use, n (%)	220 (47.9%)	71 (45.5%)	0.91
Cross clamp duration, min	140.3 (133.1 – 147.5)	139.1 (133.9 – 146.1)	0.87

Data are presented as mean (95% confidence interval) unless otherwise indicated.

Abbreviations: AKI, acute kidney injury; BMI, body mass index; eGFR, estimated glomerular filtration rate; CABG, coronary artery bypass grafting; CPB, cardio-pulmonary bypass; min, minutes.
