# Supplementary material for: Association of gain-of-function EPHX2 polymorphism Lys55Arg with acute kidney injury following cardiac surgery
Source: PLoS One. 2017 May 26;12(5):e0175292. doi: 10.1371/journal.pone.0175292 (PMC5446112; doi:10.1371/journal.pone.0175292)
Supplement: S3 Table — Data are presented as median (interquartile range) unless otherwise indicated. (PDF) [file pone.0175292.s003.pdf]

S3 Table. Baseline plasma DiHOME/EpOME ratio, a measure of soluble epoxide hydrolase activity, and epoxyeicosatrienoic acids (EETs) concentrations in patients who did or did not develop acute kidney injury (AKI)

Molar ratio	AKI<br>N=6	No AKI<br>N=25	p-value
9,10- DiHOME/EpOME	1.35 (1.10-2.63)	1.05 (0.77-1.35)	0.17
12,13-DiHOME/EpOME	1.78 (1.04-3.26)	0.98 (0.69-1.48)	0.14
Total-DiHOME/EpOME	1.41 (0.98-2.83)	1.03 (0.75-1.39)	0.18
8,9-EET, ng/mL	0.52 (0.44-0.73)	0.58 (0.42-1.19)	0.51
11,12-EET, ng/mL	0.26 (0.23-0.30)	0.50 (0.34-0.76)	0.01
14,15-EET, ng/mL	0.47 (0.37-0.60)	0.50 (0.35-0.81)	0.57
Total-EET, ng/mL	1.33 (1.15-1.47)	1.72 (1.24-2.56)	0.23

Data are presented as median (interquartile range) unless otherwise indicated.
